# Dominant Cone Rod Dystrophy, Previously Assigned to a Missense Variant in *RIMS1*, Is Fully Explained by Co-Inheritance of a Dominant Allele of *PROM1*

**DOI:** 10.1167/iovs.63.9.14

**Published:** 2022-08-10

**Authors:** Maria Pilar Martin-Gutierrez, Elena R. Schiff, Genevieve Wright, Naushin Waseem, Omar A. Mahroo, Michel Michaelides, Anthony T. Moore, Andrew R. Webster, Gavin Arno, for the Genomics England Research Consortium

**Affiliations:** 1 Moorfields Eye Hospital, London, United Kingdom; 2 UCL Institute of Ophthalmology, London, United Kingdom; 3 Department of Ophthalmology, University of California, San Francisco, San Francisco, California, United States; 4 North Thames Genomic Laboratory Hub, Great Ormond Street Hospital for Children NHS Foundation Trust, London, United Kingdom

**Keywords:** RIMS1, PROM1, CORD7, autosomal dominant cone rod dystrophy

## Abstract

**Purpose:**

Autosomal dominant cone rod dystrophy 7 (CORD7) was initially linked to the gene *RIMS1* and reported in a 4-generation British family in 1998. The purpose of this study was to investigate the legitimacy of this association, and to correctly characterize the genetic cause of this condition.

**Methods:**

The allele frequency of *RIMS1* c.2459G>A, p.Arg820His, was investigated in the Genomes Aggregation Dataset (gnomAD) datasets and whole genome sequencing (WGS) was performed for 4 members of the CORD7 family with filtering of rare pathogenic variants in a virtual gene panel comprising all genes known to be associated with inherited retinal dystrophy (IRD). Cytogenetic analysis was performed to rule out interchromosomal translocation.

**Results:**

*RIMS1* p.Arg820His has a maximal carrier frequency of >1:5000 in Europeans. A previously well-characterized *PROM1* variant: c.1118C>T, p.Arg373Cys, was detected in 9 affected members of the CORD7 family who underwent WGS or direct sequencing. One affected family member is now known to have macular dystrophy in the absence of *RIMS1* p.Arg820His. Clinical analysis of affected family members and 27 individuals with retinopathy associated with the same – *PROM1 –* variant showed consistent phenotypes.

**Conclusions:**

The case for pathogenicity of *RIMS1* p.Arg820His is not strong based on its presence on 10 alleles in the gnomAD dataset and absence from additional CORD affected individuals. The finding of a known pathogenic variant in *PROM1* correlates well with the phenotypic characteristics of the affected individuals, and is likely to account for the condition. Clear evidence of association between *RIMS1* and a retinal dystrophy is yet to be described.

Over 270 genes have so far been associated with inherited retinal dystrophies (IRDs) (RetNet: https://sph.uth.edu/retnet) with many of these associations established prior to the widespread application of massively parallel sequencing and availability of large-scale genomic datasets. These two resources have driven discovery in the field of IRD over the past decade. In order to fully refute false attributions of pathogenicity to particular genetic variants, it is important to identify the correct pathogenic variant in the original cases.^1^^–^^3^

Clinical and genetic data for an autosomal dominant cone-rod dystrophy (adCRD, CORD7) affecting 8 individuals of a 4-generation white British family (Fig. 1) were published in 1998.^4^ All affected members experienced reduction in visual acuity and dyschromatopsia between the ages of 20 and 40 years. Most individuals showed progressive deterioration in central vision over time; other symptoms included peripheral visual field constriction, difficulties seeing in bright light, and some nyctalopia in one patient. There was phenotypic variability among affected members, but all affected individuals presented with retinal changes. These included mild retinal pigment epithelium (RPE) disturbance, extensive retinal atrophy and pigmentation, attenuated retinal vessels, and bull's eye maculopathy. Fundus autofluorescence imaging in affected members showed reduced autofluorescence in the central macula, surrounded by a ring of hyperautofluorescence. Electrodiagnostic tests revealed an abnormal pattern electroretinogram (PERG) consistent with macular dysfunction, and abnormal cone and rod responses in the full field electroretinogram (ffERG) in affected individuals (although only marginally abnormal in 3 mildly affected individuals). In most affected cases, the extent of reduction in cone ERG amplitudes was greater than that seen in rod ERGs. Progressive deterioration of ffERG was seen in one individual who underwent recordings 4 years apart. The electro-oculogram (EOG) showed a normal light rise.^4^^–^^6^

**Figure 1.**
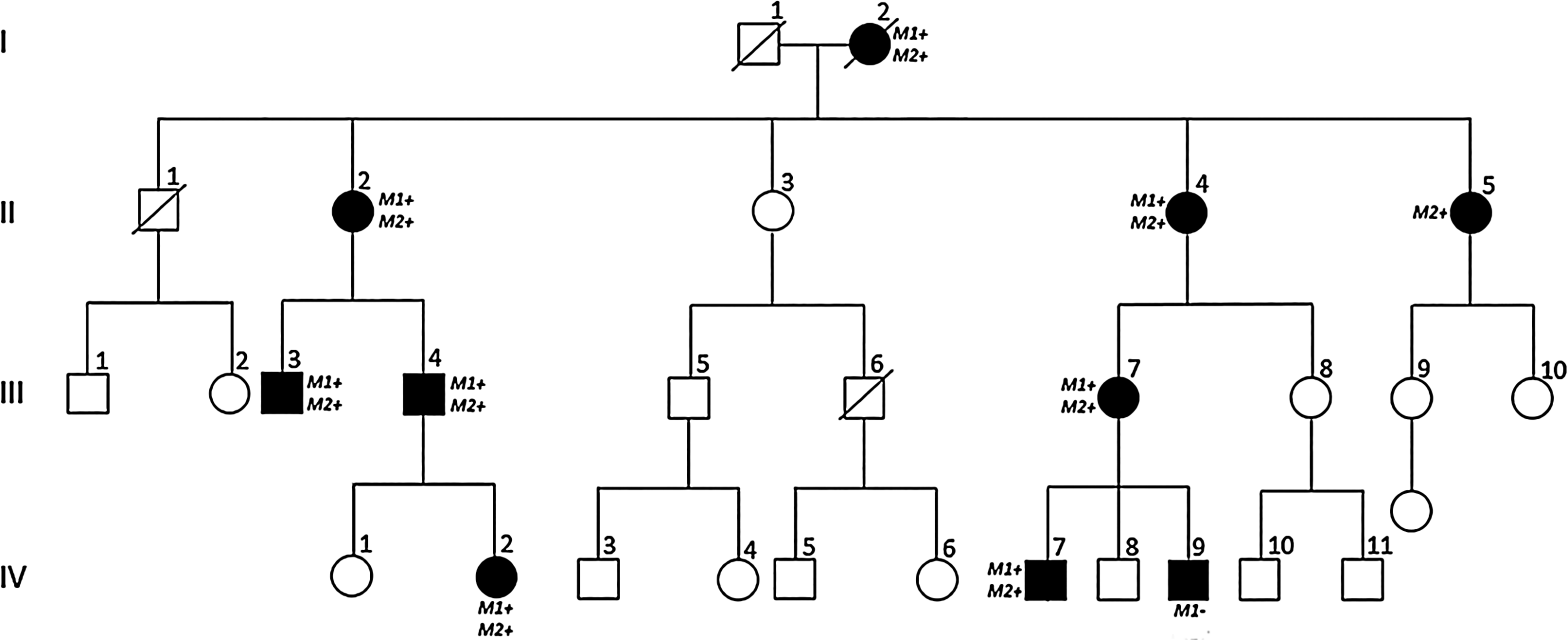
Four-generation pedigree of the British CORD7 family. Family members affected with autosomal dominant CRD are shaded. Individuals known to harbour the *RIMS1* variant p.Arg820His are labelled as “*M1*+”, and those found to carry the *PROM1* variant p.Arg373Cys, as “*M2*+”. One affected individual (IV:9) has a clinical diagnosis of macular dystrophy in the absence of the *RIMS1* variant.

Molecular genetic studies in the family established linkage to 6q14 and Sanger sequencing of *RIMS1*, a positional candidate gene, revealed a missense variant in exon 15 (c.2459G>A, p.Arg820His) which was present in 6 affected, and absent in 3 unaffected, family members. *RIMS1* is expressed in the retina, the missense change occurred in a highly conserved region of the protein and was absent in 115 ethnically matched controls. This was cited as evidence that this variant was causative.^5^ However, with the exception of one simplex case of retinitis pigmentosa (RP) found to carry heterozygous p.Arg820His *RIMS1* variant,^7^ there are no other reports of cases with retinal dystrophy associated with this variant in *RIMS1.* Furthermore, no pathogenic *RIMS1* variants have been seen in the next generation sequencing of a large cohort of patients with inherited retinal dystrophies performed at our institution and in the Genomics England “100,000 Genomes” dataset.^8^ This led us to reinvestigate the family using modern molecular genetic techniques.

## Materials and Methods

The allele frequency of GRCh37 (hg19) chr6:72,960,710G>A, NM_014989.7: *RIMS1* c.2459G>A, p.Arg820His was investigated in the Genomes Aggregation Database (gnomAD) datasets. One member of the CORD7 family was recruited for whole genome sequencing (WGS) as part of the UK 100K genomes project (100KGP).

Rare (MAF <0.001), coding variants in a virtual gene panel (http://panelapp.genomicsengland.co.uk) were interrogated for candidate pathogenic variants associated with disease in the family as part of the clinical diagnostic pipeline.

Cytogenetic analysis was also performed in one affected individual to investigate the possibility of interchromosomal translocation at the North Thames Genomic Laboratory Hub, Great Ormond Street Hospital for Children NHS Foundation Trust, London, UK, using standard protocols.

## Results

Ten carriers of *RIMS1* p.Arg820His were found in the gnomAD version 2.1 comprising 108,049 individuals and 9 carriers in the gnomAD version 3.1.1 genome dataset (34,003 individuals), with a maximal allele frequency in Europeans of 0.0001323. This carrier frequency of >1 in 5000 is considered too high for a fully penetrant dominant vision loss variant. In addition, no unrelated carriers exist in the Moorfields Eye Hospital genetic eye disease cohort of molecularly tested individuals.^8^

Whole-genome sequencing revealed that one affected member was heterozygous for a well-characterized dominant macular dystrophy/CORD allele, GRCh37 (hg19) Chr4:16,014,922G>A NM_006017.3: *PROM1* c.1118C>T p.Arg373Cys, located on chromosome 4p.^9^ Subsequent family screening confirmed that all available affected members harboured this heterozygous *PROM1* variant (see Fig. 1).

One family member, now known to have macular dystrophy, was previously reported not to harbor the *RIMS1* p.Arg820His variant, but was unavailable for *PROM1* testing (see Fig. 1, IV:9).

Given the previous segregation of the *RIMS1* p.Arg820His variant with disease in CORD7 family members and significant logarithm of the odds (LOD) score, we sought to investigate the possibility of co-segregation of a region on 6q and 4p through an interchromosomal translocation. This was excluded by interrogation of the MANTA call data from the 100KGP bioinformatics pipeline and direct interrogation of the chimeric and supplementary read alignment data and split read data. In addition, cytogenetic analysis did not show evidence of a translocation.

**Figure 2.**
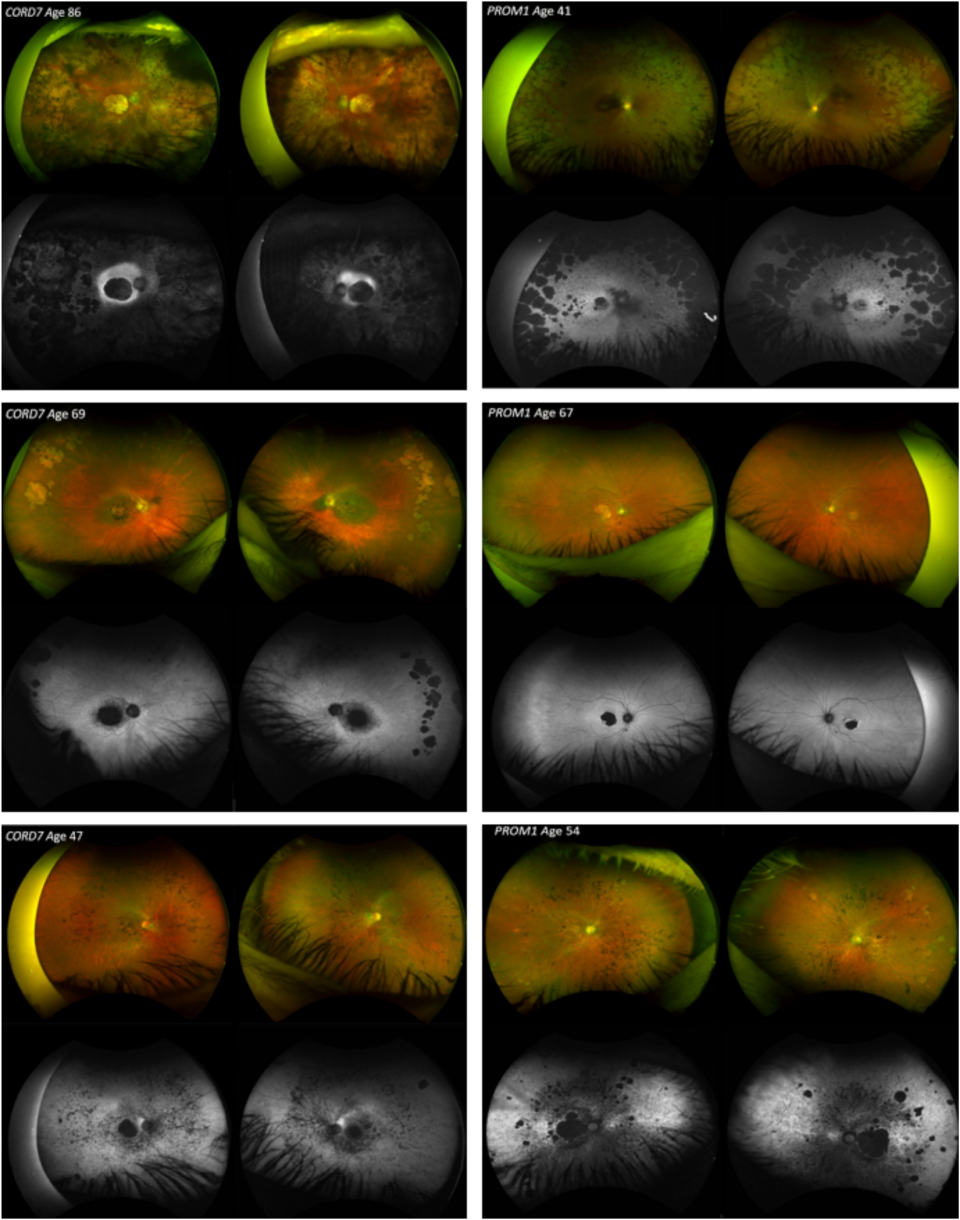
Fundoscopic features of 3 affected members of the family originally diagnosed with CORD7 (left column). Retinal changes among the affected individuals included mild maculopathy retinal pigment epithelium (RPE) changes, extensive macular and peripheral atrophy, peripheral pigmentation, bull's eye maculopathy and attenuation of retinal vessels. Note the significant intrafamilial phenotypic variability within these three images. Adjacent to each of the images, are 3 known cases of *PROM1* macular dystrophy, unrelated to the CORD family (right column). Note the similar clinical appearance with the cases shown on the left.

We surveyed the Moorfields Eye Hospital molecularly confirmed patient database^8^ and identified 27 carriers of the same *PROM1* variant with a molecular diagnostic report confirming this to be the pathogenic variant responsible for the retinal dystrophy. Review of clinical data of age-matched *PROM1-*retinopathy cases and the CORD7 affected family members showed striking similarities and a phenotype entirely consistent with *PROM1-*retinopathy in the CORD7 family (Fig. 2). The phenotype was also consistent with cases with this variant reported in the literature from References 10 and 11.

## Discussion

The case for causality of the *RIMS1* variant (p.Arg820His) has never been strong. No similarly affected unrelated individuals exist in our own cohort or in the literature. In addition, no other compelling evidence for pathogenic variants in *RIMS1* have been identified to date.^12^^–^^14^ The only other report in the literature of the p.Arg820His variant was seen in a simplex patient with RP and a discordant phenotype.^7^

The gnomAD dataset revealed that the allele frequency for the *RIMS1* p.Arg820His variant, although rare, was too common to be considered a pathogenic variant responsible for a severe vision loss disease with an onset of the first to the fifth decades (CORD7 family). Taken together, this suggests that this variant is not causative of retinal disease.^1^

*PROM1* encodes a pentaspan transmembrane domain glycoprotein that is expressed at the base of rod and cone outer segments and is involved in disc morphogenesis, photopigment sorting, and also regulation of autophagy in RPE cells. Heterozygous missense disease-causing variants in this gene cause dominant retinopathy, exhibiting a variable phenotype and variable expressivity.^10^ One such variant, *PROM1* p.Arg373Cys, has been previously reported in many cases across the literature, on different haplotypic backgrounds and is known to cause a wide spectrum of disease, including bull's eye maculopathy, macular dystrophy, and cone-rod dystrophy.^10^^,^^15^

*PROM1* p.Arg373Cys is absent from the gnomAD dataset. In contrast to the *RIMS1* variant, the *PROM1* variant is highly enriched in patients with macular dystrophy and CORD^9^ and submitted 16 times to ClinVar (15 pathogenic and 1 likely pathogenic, accessed March 2021). There are 27 carriers of the *PROM1* variant within the Moorfields patient database, and the phenotype of the CORD7 affected individuals is in keeping with the variable macular/CORD disease seen in other *PROM1*-retinopathy cases.^15^ We therefore suggest that the retinal dystrophy observed in the CORD7 family is entirely accounted for by the pathogenic variant identified in *PROM1.*

Given the robust 2-point LOD score for the 6q14 locus (3.61), we hypothesized that a translocation between chr4 and chr6 could have led to the co-inheritance of the 2 variants, but we could find no evidence of this. Reviewing the works of Kelsell et al. and Johnson et al. in the discovery papers, chr6 was considered as the first candidate for linkage with the subsequent identification of *RIMS1* (retinal expressed) as a candidate gene found to harbor a rare protein altering variant.^4^^,^^5^ This may be a “perfect storm” of coincidence and is likely therefore that this represents an alpha-error (a significant LOD score of 3.0 means there is a 1 in 1000 possibility of the trait not being linked to the locus) leading to inclusion of the gene on gene screening panels worldwide. We report the findings of macular dystrophy in one family member thought previously to be unaffected in the absence of the *RIMS1* variant, providing the first evidence of non-segregation of *RIMS1* with disease in the family.

It remains possible that variants of *RIMS1* are associated with a dominant disease given the probability of loss-of-function intolerance (pLI) of the gene is 0.99 (0–1 scale) in the gnomAD dataset suggesting that haploinsufficiency is not tolerated, and that murine knockout exhibits a complex neurological phenotype.^16^ However, no proven cases of inherited disease consequent upon loss of function variants exist to date,^12^^–^^14^ to our knowledge, and no cases of confirmed *RIMS1* disease are known. In the original publication, affected members of the study also showed enhanced cognition and this may be consequent on the p.Arg820His variant *in RIMS1*^17^ and that co-inheritance of these two phenotypes is coincidental in the family.

In conclusion, our findings suggest that the entity, previously known as CORD7, corresponds in fact to *PROM1*-retinopathy, and is consequent upon the missense pathogenic variant p.Arg373Cys. The carrier frequency of the originally reported *RIMS1* variant is higher than expected for a rare autosomal dominant disease and no other convincing cases exist in the literature. Thus, despite previous reports in the literature, there is no strong evidence to date to suggest that perturbation of *RIMS1* has a pathogenic effect on the retina in humans.
